# Free-Floating Thrombus of the Aorta: A Rare Complication of COVID-19-Induced Hypercoagulability

**DOI:** 10.7759/cureus.58676

**Published:** 2024-04-21

**Authors:** Kamal Shaik, Atika Nayeem, Rayan Ahmed, Wishwdeep Dhillon

**Affiliations:** 1 Medicine, Drexel University College of Medicine, Philadelphia, USA; 2 Internal Medicine, Mercy Gilbert Medical Center, Gilbert, USA; 3 Science, Hamilton High School, Chandler, USA; 4 Oncology, Virginia G. Piper Cancer Care Network, Gilbert, USA

**Keywords:** aortic mobile thrombus, hypercoagulable state, covid 19, thrombosis, anticoagulation

## Abstract

Free-floating thrombus (FFT) of the aorta is a rare condition characterized by a nonadherent portion of thrombus floating within the aortic lumen. Hypercoagulability is a well-known complication of COVID-19 infection, and thromboses related to COVID-19-related hypercoagulability commonly present in the form of venous or arterial thrombosis such as deep vein thrombosis (DVT), pulmonary embolism (PE), ischemic stroke, and myocardial infarction. Unfortunately, FFT associated with COVID-19 infection has been rarely reported in the literature. We report the case of a 53-year-old female patient with an unusual presentation of a pedunculated thrombus in the descending thoracic aorta caused by COVID-19-related hypercoagulability. The patient was treated with anticoagulation therapy and did not require invasive procedures. FFT is a rare but potentially catastrophic complication of COVID-19 infection. Rapid diagnosis and treatment are vital to prevent complications like limb ischemia and stroke.

## Introduction

Free-floating thrombus (FFT) of the aorta is a rare condition characterized by a nonadherent portion of thrombus floating within the aortic lumen. Clinical presentation of FFT ranges from asymptomatic disease to symptoms related to embolization of the brain and peripheral organs [1]. FFT can be a result of hypercoagulability caused by malignancy, prior surgery, or turbulent blood flow and has an approximately 75% risk of distal embolization [1,2]. Clinical factors that determine the management of FFT include thrombus size, location, mobility, and any prior history of thromboembolism [3,4]. FFT treatment options include anticoagulation, thrombectomy, thrombolysis, and endovascular grafting [5,6]. Although the link between COVID-19 infection and thromboembolism has been well established, where COVID-19 infection induces a hyperinflammatory immune response with concomitant hypoxia and diffuse intravascular coagulation that predisposes the body to thromboembolic events, COVID-19-associated FFT is uncommon [7]. Thus, we report the case of a 53-year-old female patient who presented with a pedunculated thrombus in the descending thoracic aorta caused by COVID-19-related hypercoagulability. We also discuss treatment options for management of this rare thrombotic complication of COVID-19 infection.

## Case presentation

A 53-year-old female with a past medical history of hyperthyroidism and macular degeneration presented to the Emergency Department with right lower chest pain, which worsened with inspiration. She was diagnosed with COVID-19 approximately two weeks before hospitalization. Her oxygen saturation ranged between 88% and 90% on room air. She denied any personal or family history of blood clots. She did not report alcohol, tobacco, or illicit drug use.

**Figure 1:**
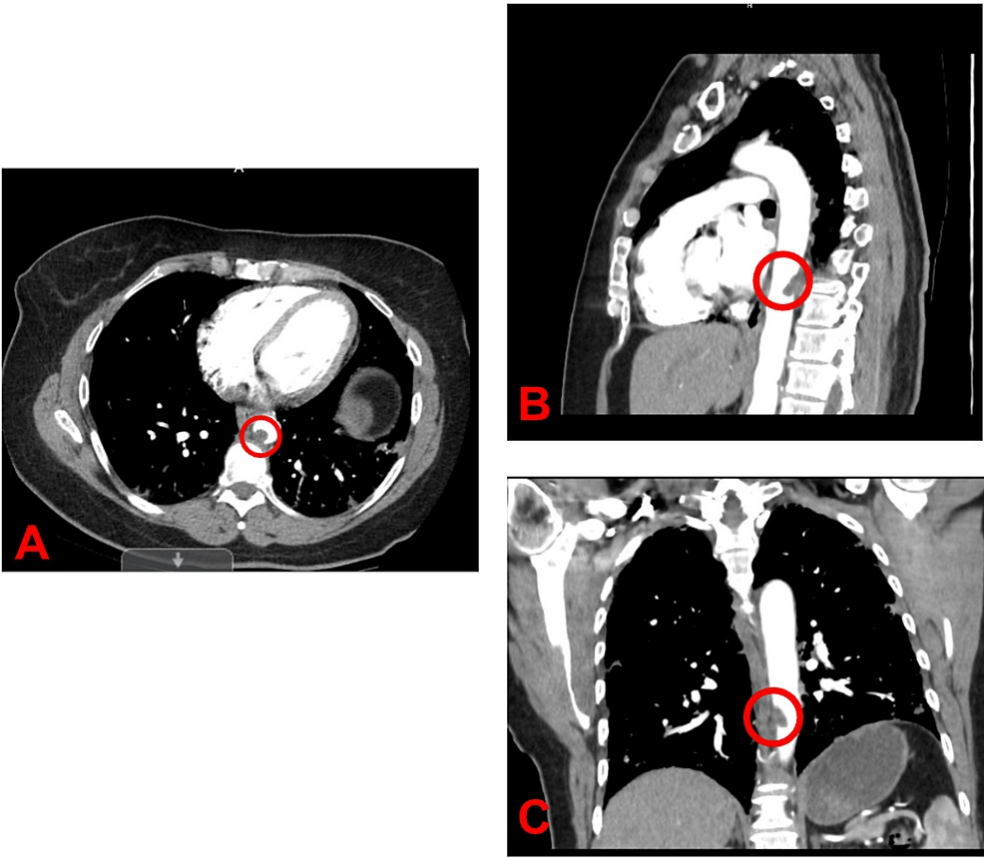
CTA of the chest showing pedunculated thrombus posteriorly in the descending thoracic aorta measuring approximately 1.7 cm in craniocaudal dimension and 1.3 cm in transverse dimension A. Axial view of CTA showing pedunculated thrombus. B. Sagittal view of CTA showing pedunculated thrombus. C. Coronal view of CTA showing pedunculated thrombus. The encircled region in A, B, and C indicates the pedunculated thrombus in the descending thoracic aorta. CT, computed tomography angiogram; AP, anteroposterior

A contrast-enhanced computed tomography (CT) of the abdomen and pelvis was positive for ground-glass opacities in the lower lobes of the lungs, typical of COVID-19 pneumonia. There was no evidence of aneurysm, dissection, or renal or mesenteric stenoses. A computed tomography angiography (CTA) of the chest was positive for a pedunculated thrombus in the descending thoracic aorta (Figure 1). Thrombophilia workup showed significantly elevated levels of anticardiolipin IgG (81 GPL U/mL) and anticardiolipin IgM (52 GPL U/mL) antibodies. Additional workup for hypercoagulability was unremarkable, including negative Factor V Leiden, lupus anticoagulant, protein C and S deficiency, and antithrombin three deficiency.

**Figure 2:**
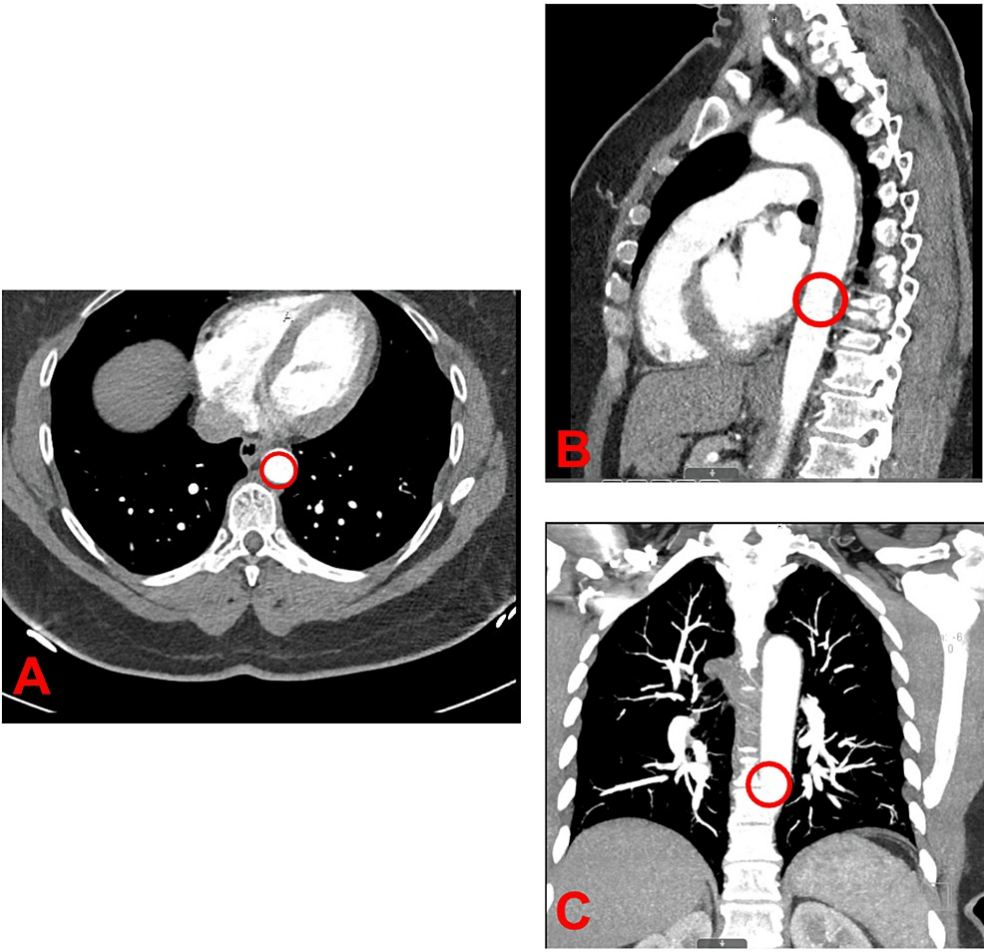
CTA of the chest post-anticoagulation treatment showing resolution of previously noted pedunculated aortic thrombus A. Axial view of CTA showing resolution of pedunculated thrombus. B. Sagittal view of CTA showing resolution of pedunculated thrombus. C. Coronal view of CTA showing resolution of pedunculated thrombus. The encircled region in A, B, and C indicates where the pedunculated thrombus was found in the descending thoracic aorta before treatment was initiated. CTA, computed tomography angiogram; AP, anteroposterior

Clinical evaluation was negative for evidence of distal embolization. A vascular surgeon evaluated her and recommended conservative management. Therapeutic anticoagulation with heparin was initiated. After discussing the risks and benefits of oral anticoagulants, she was switched to Apixaban 10 mg PO twice daily for one week, followed by 5 mg twice daily dose. After one month of anticoagulation, D-Dimer and anticardiolipin antibodies had normalized. CTA of the chest after three months of anticoagulation showed complete resolution of FFT of the descending aorta (Figure 2). The patient had an uneventful course during treatment, without any recurrent thromboembolism or bleeding. Anticoagulation was stopped after six months.

## Discussion

COVID-19 infection leads to a hypercoagulable state caused by homeostatic derangement due to the body's hyperinflammatory response [8]. Specifically, systemic cytokine production leads to platelet activation and interaction with neutrophils, creating neutrophil extracellular traps that further stimulate thrombin production, ultimately leading to thrombosis [9]. Our patient had FFT of the descending aorta, most likely caused by COVID-19-related hypercoagulability. Often, patients with a COVID-19 infection who present in a hyperinflammatory state also present with elevated levels of D-dimer, factor VIII fibrinogen, PT/aPTT, IL-6, TNF-alpha, and IL-1beta [10,11]. Additionally, numerous reports have indicated the presence of antiphospholipid antibodies (aPL Ab), predictors of hypercoagulability caused by antiphospholipid syndrome (APS), in COVID-19 infections. APS is defined by clinical criteria (thrombosis and pregnancy loss) and laboratory criteria (persistent presence of lupus anticoagulant and elevation of aPL Ab). However, it is uncertain whether an elevation of aPL Ab is an epiphenomenon or is involved in the pathogenesis of COVID-19-related hypercoagulability [12]. In this case, since the elevation in aPL Ab was transient and did not alter the duration of anticoagulation, our patient did not meet the criteria for APS.

The relative paucity of published material indicates that FFT of the aorta is an uncommon thrombotic complication, usually associated with hypercoagulable states and atherosclerosis [13]. Due to FFT's rarity, consensus regarding diagnostic criteria is lacking. Nevertheless, due to the high risk of systemic embolization in FFT, rapid diagnosis and treatment are critical. Transesophageal echocardiography (TEE) and CTA are often used to diagnose FFT [14]. Although TEE can provide high-resolution images, it cannot clearly visualize portions of the aorta, such as the ascending and upper descending aorta, which makes CTA the recommended test for diagnosis of FFT due to its rapid acquisition and high sensitivity [14,15]. Systemic anticoagulation remains the hallmark of FFT management. Thrombectomy and endovascular grafting are acceptable treatment options for patients with or at an increased risk of embolization [16,17]. The optimal duration and choice of anticoagulation remain undefined; this presents a limitation of this report, as sparse pieces of medical literature were available to guide the treatment plan’s development. Fortunately, our patient responded well to conservative management with Apixaban, as opposed to procedural management with thrombectomy or endovascular grafting, and a complete thrombus resolution was radiographically documented after three months of anticoagulation. Anticoagulation was stopped after six months because COVID-19-related hypercoagulability was deemed to be a transient risk factor for provoked thrombosis. She remained free of recurrent thrombosis after six months of stopping anticoagulation.

## Conclusions

Hypercoagulability is one of the most significant clinical phenomena associated with COVID-19 infection. Multiple studies have shown a link between COVID-19 infection and thrombotic complications like pulmonary embolism (PE), deep venous thrombosis (DVT), myocardial infarction, and stroke, among others. Management of thrombosis is a priority in patients with COVID-19 infection. Although optimal management of FFT remains undefined, systemic anticoagulation is the hallmark of treatment. Due to the high risk of embolic complications, some patients may need surgical interventions like thrombectomy and endovascular grafting. Rapid evaluation, diagnosis, and treatment are critical for avoiding embolic complications in patients with FFT. Ultimately, the purpose of this case report was to shed light on the pathophysiology, diagnosis, and management of FFT, a rare but potentially catastrophic complication of COVID-19-related hypercoagulability. In this report, the thrombus was promptly diagnosed using CTA and successfully treated with systemic anticoagulation; this report, thus, contributes to the current medical literature and future clinical treatment of FFT by supporting the usage of CTA to diagnose FFT as well as anticoagulants to resolve this condition.
